# Synchronous BALT Lymphoma and Squamous Cell Carcinoma of the Lung: Coincidence or Linkage?

**DOI:** 10.1155/2013/420393

**Published:** 2013-02-28

**Authors:** Anastasia Oikonomou, Emanuelle Astrinakis, Ioannis Kotsianidis, Vassiliki Kaloutsi, Vassileios Didilis, Konstantinos Tsatalas, Panos Prassopoulos

**Affiliations:** ^1^Department of Radiology, University Hospital of Alexandroupolis, Democritus University of Thrace, Dragana, 68100 Alexandroupolis, Greece; ^2^Department of Hematology, Democritus University of Thrace, Dragana, 68100 Alexandroupolis, Greece; ^3^Department of Pathology, Aristotelian University of Thessaloniki, 54124 Thessaloniki, Greece; ^4^Department of Cardiothoracic Surgery, Democritus University of Thrace, Dragana, 68100 Alexandroupolis, Greece

## Abstract

A 72-year-old man presented with weight loss, fever, and malaise. Chest radiograph and CT revealed two large ill-defined masses in middle and left lower lobes. CT-guided biopsy of left lower lobe mass disclosed bronchus-associated lymphoid tissue (BALT) lymphoma. Middle lobe mass was considered second deposit in contralateral lung. The patient received chemotherapy for BALT. Followup CT disclosed regression of left lower lobe mass and stability of middle-lobe mass and of right paratracheal lymph nodes. CT-guided biopsy of middle-lobe mass revealed squamous cell lung carcinoma. Surgical biopsy of right paratracheal lymph nodes revealed malignancy. Disease was staged T3, N2, and M0. Combined chemotherapy for lung cancer and BALT lymphoma was initiated.

## 1. Case Presentation

**Figure 1:**
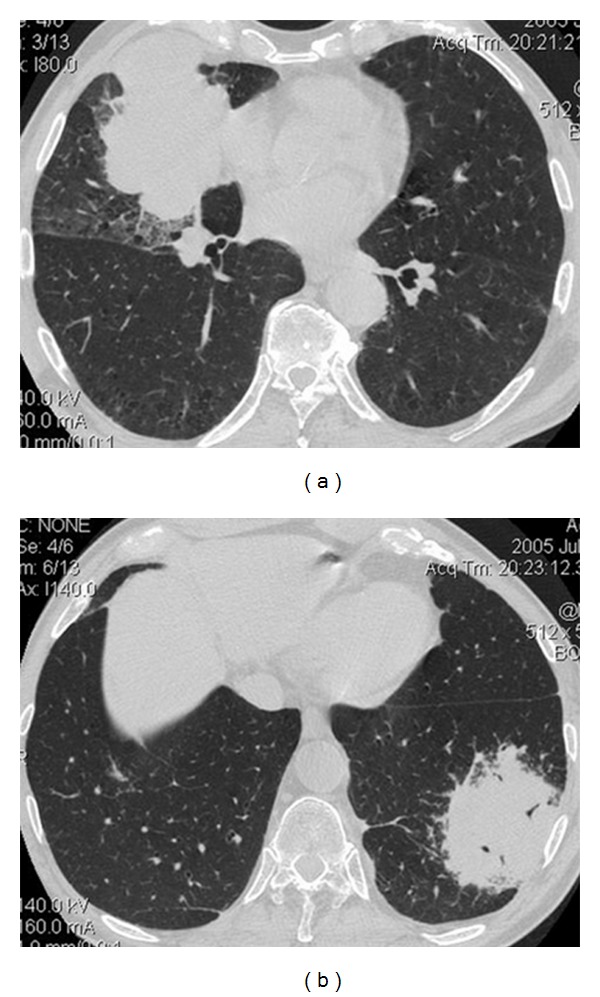
Chest CT on lung window settings shows a lobulated ill-defined mass surrounded by ground-glass opacity in the middle lobe (a) and an ill-defined masslike opacity with air bronchogram centrally in the left lower lobe (b).

**Figure 2:**
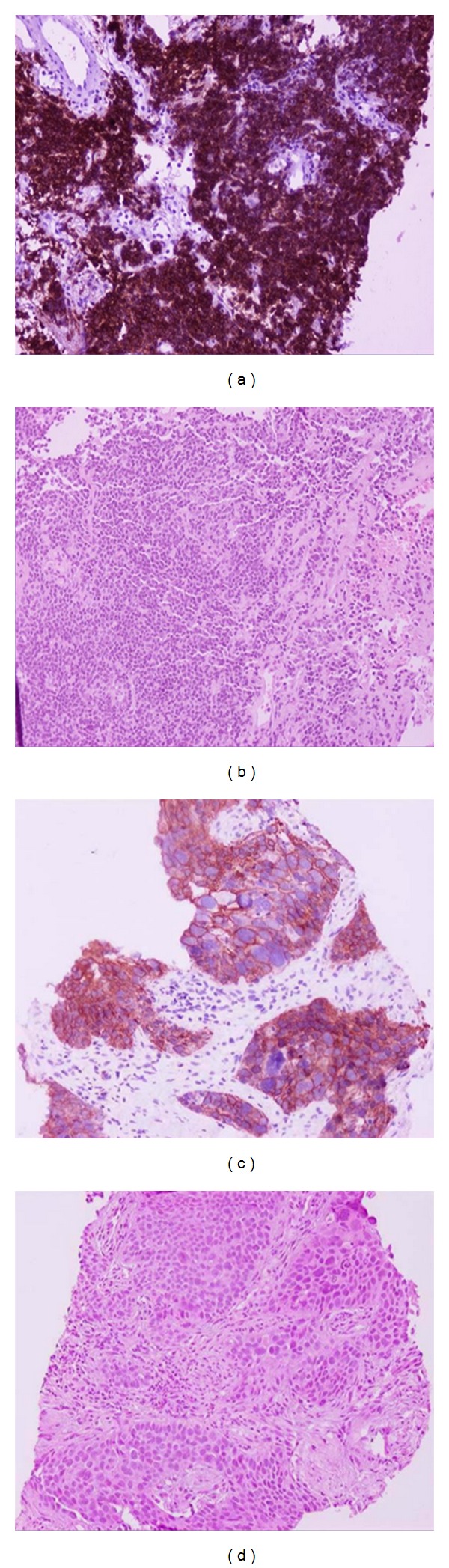
CT-guided histologic biopsy of the left lower lobe mass on immunohistochemical stain CD20 ×200 (a) and on H-E × 200 (b) reveals B-cell, mucosa-associated, non-Hodgkin lymphoma of the BALT type. CT-guided histologic biopsy of the middle lobe mass on immunohistochemical stain cyto34BE 12 × 200 (c) and on H-E × 200 (d) discloses lung cancer from squamous epithelium.

A 72-year-old man, smoker (20 pck/yr), presented with weight loss, fever, and malaise. Chest radiograph revealed two opacities in both lungs. CT of the chest verified the presence of an 8 cm ill-defined mass with lobulated margins, of homogeneous soft tissue density, surrounded by ground-glass opacity in the middle lobe, and a second 7 cm intrapulmonary soft tissue density mass in the left lower lobe with indistinct margins, exhibiting an air bronchogram (Figures 1(a) and 1(b)). A few small (<1 cm) right paratracheal lymphnodes were also noted. The left lower lobe mass was chosen for CT-guided bio n psy, as the patient would have to be positioned prone, which is more easily accepted (by the patient) as compared to the supine position. Histology disclosed bronchus associated lymphoid tissue (BALT) lymphoma (Figures 2(a) and 2(b)). The middle lobe mass was initially considered as second deposit of the same pathologic entity (BALT) in the contralateral lung. Bone marrow biopsy revealed no lymphocytic infiltration. Ann Arbor stage IIE was diagnosed. Patient's work-up for connective tissue disease was negative. The patient received 3 cycles of R-CHOP chemotherapy (monoclonal antibody rituximab, and the drugs: cyclophosphamide, doxorubicin, vincristine and prednisolone) without any side effects. The 3-month midtreatment followup CT disclosed significant size reduction of the left lower lobe mass (denoting regression of lymphoma) and stability of the middle lobe mass and of the right paratracheal lymphnodes. A CT-guided biopsy of the middle lobe mass followed and histology revealed low-differentiated squamous cell lung carcinoma (Figures 2(c) and 2(d)). Surgical biopsy of right paratracheal lymphnodes was positive for malignancy, and the disease was staged as T3, N2, and M0 and therefore combined chemotherapy for lung cancer and BALT lymphoma was initiated. Followup CT at 3 months demonstrated slight decrease in size of the middle-lobe mass which remained stable until a 9-month CT followup revealed progression of the lung cancer with distant metastatic disease. 

## 2. Discussion

Squamous cell lung cancer is the second most common histologic type of lung cancer following lung adenocarcinoma and comprising about 30% of lung cancers [1]. Bronchus-associated lymphoid (BALT) tissue in the lungs is the most common type of primary pulmonary lymphoma [2]. The presented case is the first one reported in the literature with synchronous BALT lymphoma of the lung and squamous cell lung cancer, in different lungs of the same patient. Reviewing the literature, we were able to find 6 case reports with synchronous BALT lymphoma and lung adenocarcinoma [3–5], one case with coexistence of lung adenocarcinoma with mantle cell lymphoma of the pleura [6], and one case with synchronous lung cancer (nonspecified) with tracheal mucosa-associated lymphoid tissue (MALT) lymphoma [7].

BALT lymphoma is a low-grade primary B-cell lymphoma that originates from bronchus-associated lymphoid tissue classified as an extranodal marginal zone B-cell lymphoma of MALT type/MALT lymphoma according to WHO classification [2]. It is the most common type of primary pulmonary lymphoma (>2/3 of cases), which is however a rare entity (1% of NHL) [2, 8]. BALT lymphomas have been associated with Sjogren's disease, chronic hypersensitivity pneumonitis, panbronchiolitis, dysgammaglobulinemia, amyloid deposits, collagen vascular diseases and AIDS [8]. They have an indolent growth and may be discovered incidentally on a chest radiograph. They have a very good prognosis with 5-year survival rate of 80% after treatment. Concurrent adenocarcinoma and MALT lymphoma have been previously documented in the stomach, where *Helicobacter pylori* infection seemed to be responsible for both diseases [3, 4]. Concurrent lung adenocarcinoma and BALT lymphoma have been scarcely reported in the literature, and there are speculations that a genetic linkage between the AP12-MALT1 fusion gene and the high incidence of trisomy 3 seen in MALT lymphoma may coexist with the development of lung adenocarcinoma [3, 4]. It remains to be investigated if there is also some genetic linkage between the development of BALT lymphoma and squamous cell lung carcinoma. Moreover, although smoking has not been clearly established as a risk factor for BALT lymphoma, increased expression of BALT in lung tissues from smokers has been reported. Therefore one could speculate that smoking—as in our case—could have been involved in the development of BALT lymphoma [8]. 

In the presented case, the initial histologic diagnosis of the left lower lobe mass as BALT lymphoma was misleading, as the imaging findings of both lesions were consistent with those described for BALT lymphoma: masslike opacity with air bronchogram and ill-defined mass surrounded by ground-glass opacity [8]. The imaging findings of BALT lymphoma may be variable and may also include single or multiple and often bilateral areas of consolidation or nodules with air bronchogram or air-bubble like lucencies and more rarely “tree-in-bud” pattern and diffuse interstitial lung disease [8]. Although the histologic diagnosis of the biopsied lesion was consistent with its imaging characteristics, histologic diagnosis (preferably through CT-guided biopsy) of both lesions should be the prompt diagnostic approach at first place, since the patient was a smoker and he had a high risk for developing lung cancer. 

The optimal treatment for BALT lymphoma is not well established. Surgical resection with or without chemotherapy seems to the first choice for single lesions. Bilateral, residual lesions or extrathoracic disease may be treated with chemotherapy. Chemotherapy or radiation therapy is also used in the rare cases of relapse [8]. In our case, as BALT lymphoma was initially considered to be located bilaterally in the two lungs, chemotherapy was deemed the best choice for treatment. 

In conclusion, the presence of concurrent ill-defined lung lesions in a smoker's lungs may raise the rare possibility of synchronous squamous cell lung cancer and lung lymphoma, especially if one of these lesions manifests as a masslike opacity with air bronchogram. Work-up should be based on initial histologic identification of both lesions in order to investigate the possibility of synchronous malignancies. 

## References

[1] Drilon A, Rekhtman N, Ladanyi M, Paik P (2012). Squamous-cell carcinomas of the lung: emerging biology, controversies, and the promise of targeted therapy. *The Lancet Oncology*.

[2] Cadranel J, Wislez M, Antoine M (2002). Primary pulmonary lymphoma. *European Respiratory Journal*.

[3] Ichihara E, Tabata M, Takigawa N (2008). Synchronous pulmonary MALT lymphoma and pulmonary adenocarcinoma after metachronous gastric MALT lymphoma and gastric adenocarcinoma. *Journal of Thoracic Oncology*.

[4] Kargi A, Gürel D, Akkoclu A, Sanli A, Yilmaz E (2010). Primary pulmonary extranodal marginal zone lymphoma/low grade B-cell lymphoma of MALT type combined with well-differentiated adenocarcinoma. *Tumori*.

[5] Chanel S, Burke L, Fiche M (2001). Synchronous pulmonary adenocarcinoma and extranodal marginal zone/low-grade B-cell lymphoma of MALT type. *Human Pathology*.

[6] Hatzibougias D, Bobos M, Karayannopoulou G (2008). A rare tumoral combination, synchronous lung adenocarcinoma and mantle cell lymphoma of the pleura. *World Journal of Surgical Oncology*.

[7] Suzuki T, Akizawa T, Suzuki H, Kitazume K, Omine M, Mitsuya T (2000). Primary tracheal mucosa-associated lymphoid tissue lymphoma accompanying lung cancer. Common tumorigenesis or coincidental coexistence?. *The Japanese Journal of Thoracic and Cardiovascular Surgery*.

[8] Imai H, Sunaga N, Kaira K (2009). Clinicopathological features of patients with bronchial-associated lymphoid tissue lymphoma. *Internal Medicine*.

